# Resistance of ground glass hepatocytes to oral antivirals in chronic hepatitis B patients and implication for the development of hepatocellular carcinoma

**DOI:** 10.18632/oncotarget.8388

**Published:** 2016-03-26

**Authors:** Hung-Wen Tsai, Yih-Jyh Lin, Han-Chieh Wu, Ting-Tsung Chang, I-Chin Wu, Pin-Nan Cheng, Chia-Jui Yen, Shih-Huang Chan, Wenya Huang, Ih-Jen Su

**Affiliations:** ^1^ Department of Pathology, National Cheng Kung University Hospital, College of Medicine, National Cheng Kung University, Tainan, Taiwan; ^2^ Institute of Clinical Medicine, College of Medicine, National Cheng Kung University, Tainan, Taiwan; ^3^ Center of Infectious Disease and Signaling Research, National Cheng Kung University, Tainan, Taiwan; ^4^ Department of Surgery, National Cheng Kung University Hospital, College of Medicine, National Cheng Kung University, Tainan, Taiwan; ^5^ National Institute of Infectious Diseases and Vaccinology, National Health Research Institutes, Tainan, Taiwan; ^6^ Department of Internal Medicine, National Cheng Kung University Hospital, College of Medicine, National Cheng Kung University, Tainan, Taiwan; ^7^ Department of Statistics, National Cheng Kung University, Tainan, Taiwan; ^8^ Department of Medical Laboratory Science and Biotechnology, College of Medicine, National Cheng Kung University, Tainan, Taiwan; ^9^ Department of Biotechnology, Southern Taiwan University of Science and Technology, Tainan, Taiwan

**Keywords:** ground glass hepatocytes, pre-S mutation, hepatitis B virus, anti-viral therapy, hepatocellular carcinoma

## Abstract

Ground glass hepatocytes (GGHs) have been shown to predict the development of hepatocellular carcinoma (HCC). Type I GGH and type II GGH harbor hepatitis B virus (HBV) pre-S1 and pre-S2 deletion mutants, respectively. Whether anti-HBV therapy can inhibit the expression of GGHs and potentially reduce HCC development is explored in this study. Two sets of liver specimens were included: the first contained 31 paired biopsy specimens obtained from chronic HBV patients receiving oral nucleos(t)ide analogue (NA) treatment; the second contained 186 resected liver tissues obtained from HBV-related HCC patients receiving surgery: 82 received NA before surgery and 104 did not. Compared with the baseline biopsy specimens, type I (P=0.527) and type II GGH (P=0.077) were not significantly decreased after 48 weeks of NA treatment in the first set of patients. In the second set, despite suppression of viral load (P<0.001) and periportal necrosis (P=0.006) in treated patients, GGH (P=0.594), cccDNA (P=0.172) and serum pre-S mutants (p=0.401) were not significantly suppressed. A significant decrease of type I (P=0.049) and type II GGH (P=0.029) could only be observed in patients after long duration of treatment (median duration: 4.3 years). In the treated patients, the persisted type II GGH remained an independent variable associated with decreased local recurrence-free survival of HCC (P=0.019) as in non-treated patients (P=0.001). In conclusion, the persistence of GGHs could explain the residual risk of HCC development under anti-HBV treatment. Therefore, intrahepatic GGHs and pre-S mutant are potential additional targets for HCC prevention in patients already receiving anti-HBV treatment.

## INTRODUCTION

The majority of hepatocellular carcinoma (HCC) cases are attributable to either hepatitis B virus (HBV) or hepatitis C virus (HCV) infection. Oral nucleos(t)ide analogues (NAs), including lamivudine, adefovir, entecavir, telbivudine, tenofovir are currently used to treat chronic HBV infection [1]. Early observations revealed that NA treatment not only significantly reduces the incidence of HCC [2] but also lowers HCC recurrence rate [3]. The reason NAs reduce the risk of HCC is not clear. The most likely explanation may be that NAs decrease the viral replication and thus decrease the liver injuries, inflammation, and regeneration. Short-term treatment could result in the suppression of viral replication and improvements of liver necroinflammation. Long-term treatment for at least 3 years could result in no worsening of the Knodell fibrosis score in most patients [4]. Although NAs are effective to suppress HBV replication and reduce HCC development, a high proportion, up to 45%, of patients still suffered from HCC recurrence after surgery despite anti-HBV therapy [3]. The intrahepatic covalently closed circular DNA (cccDNA) was found to persist after oral anti-viral NA [5], explaining the persistence of HBV infection.

To guide future anti-viral treatment and HCC prevention, it is important to understand the response of intrahepatic viral status and liver pathology to current anti-HBV treatment. Recently, ground glass hepatocytes (GGHs) harbor pre-S deletion mutants have been reported to represent the pre-neoplastic lesions and can predict the development of HCC [6–9]. There are two types of GGHs. Type I GGHs contain pre-S1 deletion mutants and usually exhibit a globular or inclusion-like HBsAg staining pattern in the cytoplasm and scatter sporadically in liver lobules; whereas type II GGHs contain pre-S2 mutants, exhibit a marginal HBsAg staining pattern in the cytoplasm and consistently cluster in groups [10]. The pre-S mutants are retained in the endoplasmic reticulum (ER) of GGH and induce ER stress-dependent response signals and HCC development [6, 7, 11, 12]. Therefore, whether antiviral therapy can effectively suppress the expression of GGHs or pre-S mutation is a critical question to be clarified. Answering this question would help us to understand the underlying mechanism of HCC development or *de novo* HCC recurrence in patients already receiving anti-viral treatment.

In this study, we evaluated the effects of anti-HBV treatment on the expression of intrahepatic GGHs using two sets of liver specimens. The first set contained paired liver biopsies from 31 patients receiving oral NA treatment. The second set consisted of resected liver specimens obtained from 186 HBV-related HCC patients receiving surgery. Besides GGHs, serum pre-S mutation status and the intrahepatic expression of cccDNA, HBV antigens, necroinflammation and fatty liver disease in the non-tumorous liver of HCC patients were simultaneously studied.

## RESULTS

### GGHs resisted NA treatment in paired biopsy samples

**Figure 1:**
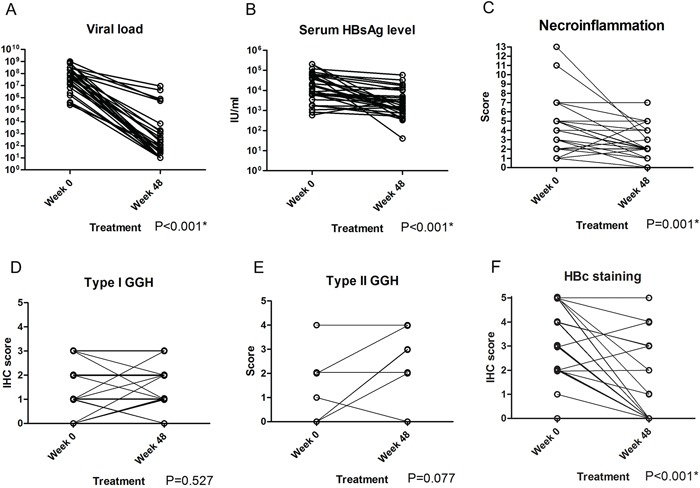
Comparisons of serum viral load **A.** serum HBsAg level **B.** Knodell necroinflammatory score **C.** type I GGH **D.** type II GGH **E.** and HBcAg staining **F.** between week 0 and week 48 of NA treatment in chronic hepatitis B patients.

**Figure 2:**
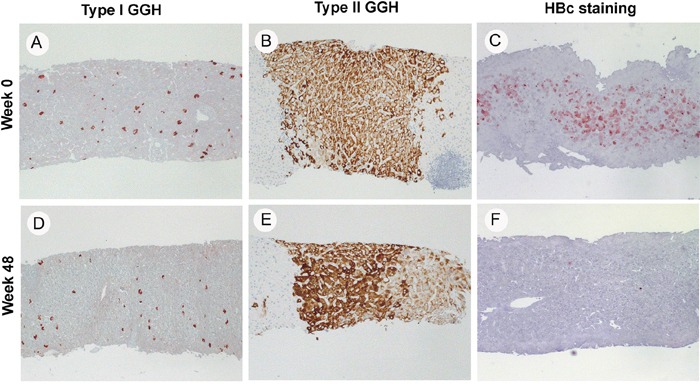
Histologic pictures of type I GGH **A.** and **D.** type II GGH **B.** and **E.** and HBc stain **C.** and **F.** in three representative cases at week 0 and week 48 of NA treatment (40X).

We first confirmed the resistance of GGH to NA treatment in patients with paired liver biopsy samples. There were 29 men and 2 women, and the mean age of all patients was 34.7 years (range: 18.4~54.3 years). Ten patients received entecavir, eight patients received adefovir and 13 patients received lamivudine. Patient profiles are summarized in Supplementary Table 1. Type I GGHs, type II GGHs and HBcAg expression were present in 48.4%, 12.9% and 87.1% of baseline biopsy specimens and 58.1%, 19.4% and 35.5% of week 48 biopsy specimens, respectively. Compared with baseline data, ALT (P<0.001), viral load (P<0.001), serum HBsAg level (P<0.001), necroinflammatory score (P=0.001) and HBcAg stain (P<0.001) were significantly improved after 48 weeks of NA treatment while type I GGH (P=0.527) and type II GGH (P=0.077) were not significantly decreased (Figure 1, Figure 2 and Supplementary Table 1).

### cccDNA and GGHs were not significantly decreased in HCC patients with pre-surgical anti-HBV treatment

We then studied the potential long-term resistance of GGHs to NA treatment in 186 HCC patients. The 186 patients were divided into anti-HBV non-treatment group (N=104) and treatment group (N=82) according to the drug history of anti-HBV agents before surgery. There was no significant difference in age, sex, HBeAg status and tumor stage between the two groups (Supplementary Table 2). In the treatment group, all cases received NA and only one patient ever received additional pegylated interferon (Peg-IFN) (Supplementary Table 3). Most of the cases (N=72) received Entecavir, either from the beginning (N=58) or following other agents (N=14). The medium duration of pre-surgical treatment was 1.65 years (range, 2.4 weeks to 11 years).

**Figure 3:**
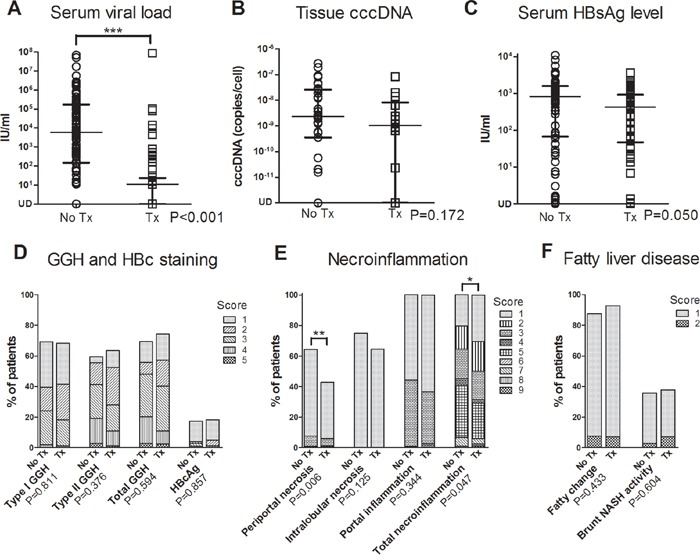
Comparisons of serum viral load **A.** tissue cccDNA **B.** serum HBsAg level **C.** and non-tumorous liver pathologic findings, including viral protein expression **D.** Knodell necroinflammatory score **E.** and fatty liver disease **F.** between non-treatment group (no Tx) and treatment group (Tx) in HCC patients. (Lines in A-C at median with interquartile range. GGH, ground glass hepatocyte; *, P<0.05; **, P<0.01; ***, P<0.001).

NAs successfully suppressed viral replication as serum viral load at the time point of surgery was significantly lower in treatment group than non-treatment group (P<0.001) (Figure 3A and supplementary Table 2). However, tissue cccDNA was not significantly suppressed (P=0.172) (Figure 3B). Serum HBsAg was marginally suppressed (P=0.050) (Figure 3C).

**Figure 4:**
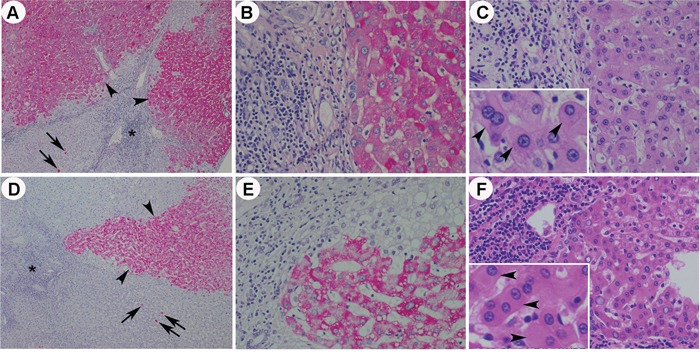
Representative histological pictures from a patient with 1.7 years of anti-HBV treatment **A~C.** and a patient with 4.3 years of anti-HBV treatment **D~F.** The low power fields (40X) of HBsAg stain showed persistent type I GGH (arrows) and type II GGH (arrow heads) in patients with treatment (A and D) (Asterisk: portal area). Higher power fields (200X) of the type II GGH in HBsAg stain (B and E) and corresponding images of the type II GGH (exemplified by arrow heads in the insets, 400X) in H&E stain (C and F) were shown.

Type I GGH, type II GGH and HBcAg expression were seen in 69.2%, 59.6% and 17.3% in non-treatment group and in 68.3%, 63.4% and 18.3% in treatment group, respectively. GGH was not significantly suppressed by NA (P=0.594, P=0.811 and P=0.376 for total GGH, type I GGH and type II GGH, respectively) (Figure 3D). Periportal necrosis score (P=0.006) and total necroinflammatory score (P=0.047) were significantly lower in treatment group (Figure 3E). Fatty liver disease also showed no significant difference between these two groups (P=0.433 and P=0.604 for fatty change and NASH activity, respectively) (Figure 3F). Representative histological pictures from a patient without NA treatment (Supplementary Figure 1), a patient with 1.7 years of treatment (Figure 4A~4C) and another patient with 4.3 years of treatment (Figure 4D~4F) showed that type I GGH and type II GGH could persist under NA treatment.

### The inhibitory effect of oral nucleos(t)ide analogue on GGHs could only be observed in patients with long duration of treatment

We investigated the duration of pre-surgical anti-HBV therapy in affecting serum markers and liver pathology. The parameters were plotted according to different years of NA treatment to estimate the treatment effects (Supplementary Figure 2). The viral load showed a significant rapid drop within first year of treatment (P<0.001) (Supplementary Figure 2A) while the cccDNA, serum HBsAg, GGH and portal inflammation showed a slow decline (Supplementary Figure 2B-F). We thus separated the treated cases into 3 groups according to the duration of pre-surgical anti-viral treatment: 25 patients had short duration of treatment for less than one year (median duration of treatment: 4.6 months, range: 0.6~10.8 months), 35 patients had medium duration of treatment for more than one year but less than 3 years (median duration of treatment: 1.7 years, range: 1~2.9 years) and 22 patients had long duration of treatment for more than 3 years (median: 4.3 years, range: 3~11 years) (Supplementary Figure 3). The tissue cccDNA and serum HBsAg level was not significantly lower in patients with longer duration of treatment (P=0.624 and P=0.686, respectively) (Supplementary Figure 3A and 3B). Type I GGH, type II GGH, total GGH and portal inflammation were significantly lower with longer duration of treatment (P=0.049, P=0.029, P=0.025 and P=0.030, respectively) (Supplementary Figure 3C and 3D). Post-hoc tests showed the significant differences present between patients with more than 3 years of treatment and those with less than 1 year of treatment (P=0.040, P=0.033, P=0.022 and P=0.042, respectively). The fatty liver disease was not significantly lower in patients with longer duration of treatment (Supplementary Figure 3E).

GGHs were not significantly associated with ALT, Child-Pugh score, albumin and total bilirubin in both non-treatment group and treatment group (Supplementary Figure 4 and 5). In the treatment group, type I GGH and II GGH were significantly associated with serum HBsAg level (P=0.006 and P=0.025, respectively). Type I GGH and type II GGH had borderline associations with cccDNA (P=0.065 and P=0.097, respectively). HBcAg staining was positively associated with viral load (P=0.035) (Supplementary Figure 6). For necroinflammation, type I and type II GGH were significantly associated with portal inflammation (P=0.002 and P=0.009, respectively). HBcAg staining was positively associated with periportal necrosis (P=0.002), intralobular necrosis (P=0.043) and portal inflammation (P=0.007) (Supplementary Figure 7).

### Pre-S mutation rate was not significantly different between non-treatment group and treatment group

Since type I GGH and type II GGH contain Pre-S deletion mutant in the liver tissue, we also investigated the pre-S mutation status in serum. Pre-S deletion mutation was detected in 35.9% of patients: 9.2% had pre-S1 mutation, 19.1% had pre-S2 mutation and 7.6% had mutations both in pre-S1 and pre-S2 region. The pre-S mutation status was not different between non-treatment group and treatment group (P=0.317, P=0.943 and P=0.401 for pre-S1, pre-S2 and total pre-S mutation, respectively) (Supplementary Figure 8A). In treatment group, pre-S mutation was detected in 46.2%, 33.3% and 14.3% of patients with less than one year, 1~3 years and more than 3 years of treatment, respectively. However, the differences were not significant statistically (P=0.859, P=0.369 and P=0.203 for pre-S1, pre-S2 and total pre-S mutation, respectively) (Supplementary Figure 8B).

### Prognostic significance of GGH patterns, HBV serum profiles and clinicopathological indicators for patients with HCC

**Figure 5:**
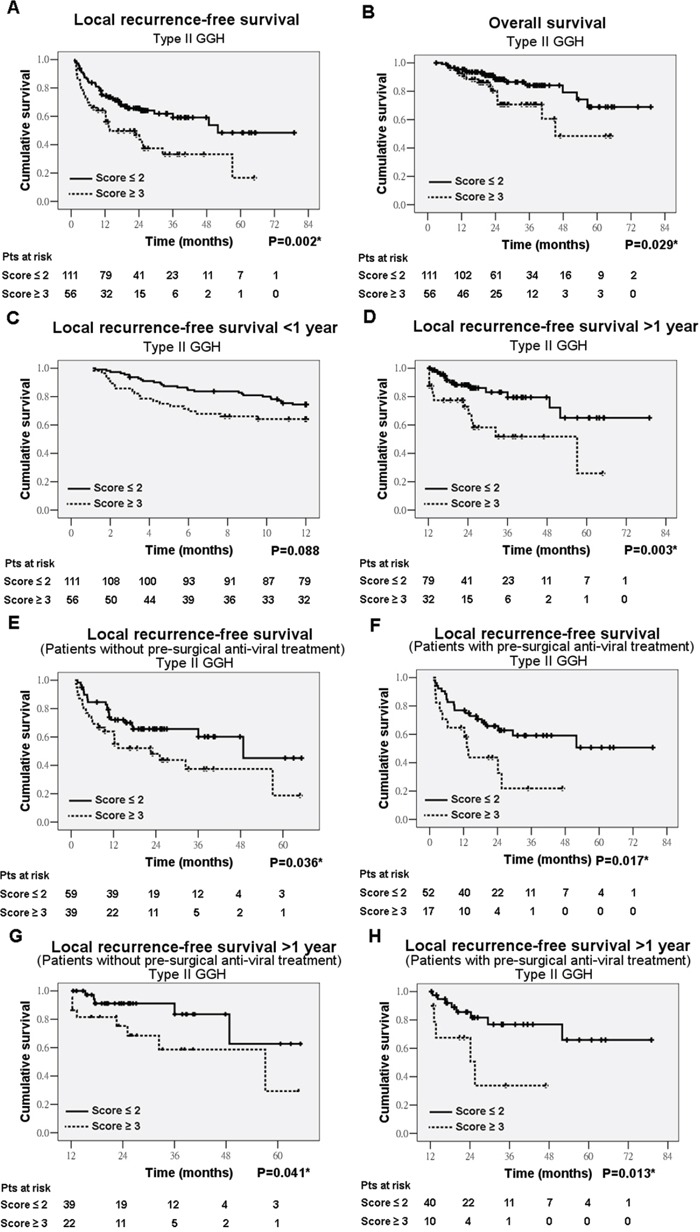
Kaplan-Meier analysis of type II ground-glass hepatocytes (GGH) in association with local recurrence-free survival (LRFS) and overall survival (OS) LRFS **A.** OS **B.** early recurrence **C.** and late recurrence **D.** in total cases. LRFS in non-treatment group **E.** and treatment group **F.** Late recurrence in non-treatment group **G.** and treatment group **H.**

Kaplan-Meier analysis in patients with complete tumor resection showed that type I GGH has a marginal significance in association with decreased local recurrence-free survival (LRFS) (P=0.099) (data not shown) and type II GGH were significantly associated with decreased LRFS (P=0.002) (Figure 5A) and decreased overall survival (OS) (P=0.029) (Figure 5B). There is no significant difference in LRFS between non-treatment group and treatment group (P=0.279). *De novo* recurrence arisen from underlying diseased liver usually occurs as late tumor recurrence [13]. The type II GGH was significantly associated with late recurrence (P=0.003) but not early recurrence (P=0.088) (Figures 5C and 5D). Multivariate analysis revealed that tumor size (P<0.001; odds ratio [OR] = 2.846; 95% confidence interval [CI], 1.643-4.928), AJCC stage (P<0.001; OR = 4.316; 95% CI, 1.901-9.797), and type II GGH (P<0.001; OR = 3.090; 95% CI, 1.752-5.171) were the independent variables associated with decreased LRFS (Supplementary Table 4). AJCC stage (P<0.001; OR = 11.857; 95% CI, 4.983-28.215), Knodell inflammatory score (P<0.001; OR = 9.808; 95% CI, 3.530-27.253) and type II GGH (P=0.022; OR = 2.350; 95% CI, 1.131-4.883) were the independent variables associated with decreased OS (Supplementary Table 4).

We further analyzed the 98 patients without pre-surgical anti-HBV drug treatment and 69 patients with pre-surgical anti-HBV drug treatment, separately. Kaplan-Meier analysis showed that type II GGH was significantly correlated with decreased LRFS in both non-treatment group (P=0.036) (Figure 5E) and treatment group (P=0.017) (Figure 5F). Further analysis showed that type II GGH was associated with late recurrence in non-treatment group (P=0.041) (Figure 5G) and treatment group (P=0.013) (Figure 5H) but not early recurrence. The GGH was not associated with OS when analyzed separately. In the non-treatment group, multivariate analysis revealed that pre-S1 mutation (P=0.001; OR = 4.553; 95% CI, 1.859-11.151), tumor size (P<0.001; OR = 4.923; 95% CI, 2.249-10.779), AJCC stage (P=0.005; OR = 4.248; 95% CI, 1.543-11.697), and type II GGH (P=0.001; OR = 4.488; 95% CI, 1.992-10.113) were the independent variables associated with decreased LRFS (Table 1). In the treatment group, multivariate analysis revealed that AJCC stage (P=0.007; OR = 4.287; 95% CI, 1.497-12.274) and type II GGH (P=0.019; OR = 2.455; 95% CI, 1.162-5.190) remained the independent variables associated with decreased LRFS (Table 1).

**Table 1:** Prognostic significance of clinicopathological indicators, GGH patterns and HBV serum profiles for the HCC patients without or with pre-surgical anti-HBV treatment

Factor	Group	LRFS Non-treatment group		LRFS Treatment group	
		Univariate	multivariate	Univariate	Multivariate
***Serum profiles***					
Viral load (IU/ml)	(<10^4^, ≥10^4^)	0.292		0.297	
HBsAg level (IU/ml)	(<800, ≥800)	0.015*	NS	0.403	
Pre-S1 mutation	(−,+)	0.047*	0.001*	0.472	
Pre- S2 mutation	(−,+)	0.140		0.783	
Pre-S1 and S2 mutation	(−,+)	0.097		0.849	
***Tumor factor***					
Differentiation	(W-M, P)	0.096		0.032*	NS
Multifocal tumors	(−,+)	0.373		0.060	
Satellite nodule	(−,+)	0.022*	NS	0.004*	NS
Size (cm)	(<5, ≥5)	<0.001*	<0.001*	0.080	
Encapsulation	(−,+)	0.497		0.692	
Vascular invasion	(−,+)	0.030*	NS	0.197	
AJCC stage	(I, ≥II)	0.011*	0.005*	0.007*	0.007*
***Non-tumor liver pathology***					
Knodell necroinflammatory score	(≤5,≥6)	0.197		0.052	
Knodell fibrosis score	(≤2,≥3)	0.192		0.522	
Fatty change	(<5%,≥5%)	0.414		0.927	
Type I GGH score	(0-2, 3-4)	0.118		0.577	
Type II GGH score	(0-2, 3-4)	0.039*	0.001*	0.021*	0.019*
HBcAg score	(0-2, 3-4)	0.022*	NS	0.012*	NS

NS, not significant; LRFS, local recurrence-free survival; OS, overall survival; (W, M, P), (well, moderately, Poorly-differentiated on the basis of the World Health Organization classification). * P<0.05.

## DISCUSSION

Antiviral treatment has been shown to reduce the incidence of HBV associated HCC (reduced from 9.7 to 3.3 per 100 person years in one meta-analysis study) [2] but there is still residual risk of HCC development despite anti-HBV therapy. Since GGH is one of the factors that are responsible for hepatocarcinogenesis, we wanted to investigate whether antiviral treatments can suppress GGH formation in patients with chronic HBV infection. We demonstrated for the first time that GGHs in the liver tissue resist to NA treatment despite suppression of HBV replication, necroinflammation and HBcAg. Most patients with paired biopsy in our study were serum HBeAg-positive and in the active viral replication with immune response stage while most patients with HCC were serum HBeAg-negative and in the low viral replication stage. Although GGHs were less prevalent in active viral replication stage, the resistance of GGHs to NA treatment was seen in both stages. The type II GGHs were even marginally increased (P=0.077) after 48 weeks of treatment in the paired biopsy cohort, suggesting that the formation of GGHs in this stage were not inhibited under NA treatment.

The intrahepatic cccDNA and serum pre-S mutation rate also showed no significant reduction under NA treatment. Moreover, a low-grade portal inflammation, indicating a chronic persistent hepatitis pattern, persisted in association with GGHs in HCC patients. GGHs and portal inflammation were only observed to significantly decrease in patients receiving long duration of NA treatment (median duration of treatment: 4.3 years). These findings suggest that the viral oncoproteins can be continuously produced in the liver for years under NA treatment, presumably using cccDNA or integrated HBV genome as the template [14, 15]. The type II GGH was significantly associated with decreased LRFS, especially late recurrence in both non-treatment group and treatment group, suggesting that the long-term resistance of GGHs may act as part of the cancer field effect and contribute to the remaining risk of HCC incidence or *de novo* HCC recurrence in patients already receiving anti-viral treatment.

Distinct from oral NAs, Peg-IFN has been shown to effectively suppress cccDNA and reduce serum HBsAg level [15, 16]. Only one case in this study cohort had been treated with Peg-IFN in addition to long-term NA (supplementary Table 3). His liver at the time of surgery contained a low level of type I GGH (score 1) and type II GGH (score 1). More cases are needed to clarify the inhibitory effect of combined nucleoside analogue and Peg-IFN treatment. Recently, lymphotoxin-β receptor (LTβR) and downstream APOBEC3 cytidine deaminase pathway has also been proposed to be an alternative mechanism to specifically degrade cccDNA [17]. Therefore, combining NA and drugs that degrade cccDNA, such as Peg-IFN and LTβR activator to accelerate GGH elimination and reduce HCC development or recurrence is worth further investigation. By observing GGH in liver tissue or developing a method to specifically measure the serum level of pre-S mutant form could help us identify a subpopulation of patients who need the combined therapy. Furthermore, the response of intrahepatic GGH could be used as a marker to evaluate the efficacy of future treatment strategy.

Compared with the relatively rapid HBV replication suppression, it is unexpected to note that a low-grade portal inflammation was observed after long duration of NA treatment in the HCC patients (Supplementary Figure 2F and 3D). HBV carcinogenesis may include two mechanisms: host anti-viral immune response and viral oncoprotein expression and insertional mutagenesis [18]. The host immune response is associated with CD8-mediated liver injury, resulting in necrosis, fibrosis and regeneration which may prevail at the early stage of HBV infection. As the disease progresses, viral oncoprotein-driven tumorigenesis pathways take place when there is low or no viral replication. The HCC cases represent late stage of natural history of HBV infection. Persistent HBcAg expression may explain only a small proportion (18.3%) of patients with necroinflammatory activity in the treatment group. Portal inflammation decreased in parallel with GGH under NA treatment. It is plausible that the persistent viral proteins, especially pre-S1 mutant in type I GGH may drive the host immune reaction in the low viral replication stage.

## MATERIALS AND METHODS

### Patients, samples and clinicopathological data

In the first set, thirty-one treatment-naïve chronic hepatitis B patients with paired liver biopsy samples at baseline and at week 48 of NA treatment at National Cheng Kung University Hospital (NCKUH) were included (Supplementary Table 1).

For treatment effect of NA on GGHs in HCC stage, the second set consisted of 186 consecutive patients who had HBV-related HCC underwent surgery at NCKUH from January 2009 to April 2014. Nine patients received liver transplantation and 177 patients received surgical resection. Patients with combined HCV infection were not included. Samples were retrieved from the Human Biobank at NCKUH. The clinicopathological records were collected including the type and duration of anti-HBV therapy before the time point of surgery. The patients were divided into anti-HBV non-treatment group and treatment group according to the drug history of anti-HBV agents before surgery (Supplementary Table 3). Serum samples that were obtained at the time point of surgery were analyzed for HBV viral load, HBsAg level and pre-S mutant status. Archival frozen or paraffin samples of non-tumorous liver parenchyma from each patient were analyzed for cccDNA, pattern of GGHs, HBsAg staining, HBcAg staining, Knodell necroinflammatory and fibrosis score [19], and fatty change. Knodell periportal necrosis score, intralobular necrosis score and portal inflammatory score were added for a total necroinflammatory score. The severity of fatty change was evaluated with grades from 0 to 3 corresponding to the percentage of fatty change in <5%, 5%~33%, 33%~66%, and >66% of the liver parenchyma. Steatohepatitis activity was evaluated by Brunt system [20]. To evaluate prognostic significances of GGH and pathologic parameters on local tumor recurrence and survival, the 9 patients with liver transplantation and 10 patients with persisted tumor after resection due to positive surgical margins were excluded. The remaining 167 patients were included. The median duration of follow-up after surgery was 24.6 months (range, 3-74.8 months). Seventy-four patients (44.3%) had local recurrence of HCC (median duration until recurrence, 8.6 months; range, 1.1-57.1 months). Thirty patients (18%) died during follow-up (median survival, 19.8 months; range, 5-56.6 months). This study was approved by the Human Experiment and Ethics Committee of NCKUH (A-BR-101-133, 2012/12/3 and B-ER-102-305, 2013/11/14).

### Immunohistochemical analysis

Immunohistochemistry was performed on 5-μm-thick formalin-fixed paraffin-embedded sections. Anti-HBsAg (T9, Neomarkers) and anti-HBcAg (LF161, Novocastra) were used as the primary antibodies. The procedures were done with the Benchmark XT autostainer (Ventana Medical Systems Inc., Tucson, USA). Deparaffinized sections were incubated with anti-HBsAg at room temperature for 16 min or anti-HBcAg at 37°C for 12 min. Subsequently, sections were visualized by an aminoethyl carbazole substrate kit (Zymed Laboratory, Inc, San Francisco, CA) or the Ultraview Universal Alkaline Phosphatase Red Detection Kit (Ventana). Counterstaining was carried out with hematoxylin. The GGH patterns in non-tumor part liver parenchyma were classified as follows: type I GGH pattern with strong, globular, or “inclusion-like” staining (Supplementary Figure 1A and 1B); and type II GGH pattern identified as hepatocytes with dense surface antigen staining at the cell margin or periphery (Supplementary Figure 1C and 1D) [8]. For HBcAg staining, both cytoplasmic staining and nuclear staining were considered positive. The percentage of immunostaining or the GGH patterns were determined manually by the consensus of 2 separate pathologists (H.-W.T. and I.-J.S.) who were blinded to the clinical outcomes at the time of review. A semi-quantitative expression scoring system modified from a previous study [8] was used with scores from 0 to 5 corresponding to the percentage of positive immunostaining in 0%, <5%, 5% to 9%, 10% to 29%, 30 to 59% and ≥60% of hepatocytes, respectively.

### HBV viral load and HBsAg quantification in serum

Serum HBV viral load was determined using the TaqMan polymerase chain reaction assay (COBAS AmpliPreP/CoBAS TaqMan HBV Test, Roche Molecular System, Branchburg, NJ [lower limit of detection, 20 IU/mL]). Serum HBsAg was quantified using the Abbott Architect HBsAg Assay (Abbott Laboratories, Sligo, Ireland) according to the manufacturer's instructions.

### Quantification of HBV cccDNA using real-time PCR

Fifty-three fresh frozen non-tumor part liver tissue samples of HCC patients were available for cccDNA analysis. HBV cccDNA quantification was performed using Roche LightCycler system. The primers and probe set were adopted from Chen Y et al [21]. The PCR program consisted of an initial denaturing step at 95 for 10 min, followed by 45 amplification cycles at 95 for 10 sec, 55 for 20 sec and at 72 for 20 sec.

### Pre-S mutation detection in serum

One hundred and thirty-one HCC patients had available serum for pre-S analysis. Serum (200ul) was used for DNA extraction with QIAamp DNA Mini kit (QIAGEN, Germany). The polymerase chain reaction (PCR), TA cloning and detection of pre-S mutation using the Pre-S Gene Chip were previously described in our study [22]. DNA sequencing for pre-S mutations was also performed to validate the chip data.

### Statistics

The differences between two paired groups were compared by using Wilcoxon signed ranks tests. The differences between two groups were compared by using Wilcoxon rank sum tests, chi-square test or Fisher's exact test as appropriate. The differences among patients with different treatment durations were compared using Kruskal-Wallis and Dunn's post-hoc tests. The correlations were calculated using Spearman correlation. The LRFS and OS rates were calculated by using the Kaplan-Meier method, and the log-rank test was used to assess the significance between groups. Cox proportional hazards regression models with a forward stepwise selection method were used to assess the independence of different factors, and only those prognostic variables that had P values <0.05 in univariate analysis were included in the model. Values of p less than 0.05 were considered statistically significant.

## CONCLUSIONS

NA treatment cannot effectively reduce the cccDNA and pre-S mutation rate and only slowly suppresses GGH which still drive HCC tumorigenesis in patients already receiving NA treatment. Therefore, combining long-term NA treatment and novel drugs to inhibit cccDNA, pre-S mutants and GGH is needed to prevent HCC development or de novo recurrence.

## SUPPLEMENTARY FIGURES AND TABLES



## References

[1] Singal AK, Fontana RJ (2012). Meta-analysis: oral anti-viral agents in adults with decompensated hepatitis B virus cirrhosis. Aliment Pharmacol Ther.

[2] Singal AK, Salameh H, Kuo YF, Fontana RJ (2013). Meta-analysis: the impact of oral anti-viral agents on the incidence of hepatocellular carcinoma in chronic hepatitis B. Aliment Pharmacol Ther.

[3] Wu CY, Chen YJ, Ho HJ, Hsu YC, Kuo KN, Wu MS, Lin JT (2012). Association between nucleoside analogues and risk of hepatitis B virus-related hepatocellular carcinoma recurrence following liver resection. JAMA.

[4] Chang TT, Liaw YF, Wu SS, Schiff E, Han KH, Lai CL, Safadi R, Lee SS, Halota W, Goodman Z, Chi YC, Zhang H, Hindes R, Iloeje U, Beebe S, Kreter B (2010). Long-term entecavir therapy results in the reversal of fibrosis/cirrhosis and continued histological improvement in patients with chronic hepatitis B. Hepatology.

[5] Cheng PN, Liu WC, Tsai HW, Wu IC, Chang TT, Young KC (2011). Association of intrahepatic cccDNA reduction with the improvement of liver histology in chronic hepatitis B patients receiving oral antiviral agents. J Med Virol.

[6] Wang HC, Huang W, Lai MD, Su IJ (2006). Hepatitis B virus pre-S mutants, endoplasmic reticulum stress and hepatocarcinogenesis. Cancer Sci.

[7] Yang JC, Teng CF, Wu HC, Tsai HW, Chuang HC, Tsai TF, Hsu YH, Huang W, Wu LW, Su IJ (2009). Enhanced expression of vascular endothelial growth factor-A in ground glass hepatocytes and its implication in hepatitis B virus hepatocarcinogenesis. Hepatology.

[8] Tsai HW, Lin YJ, Lin PW, Wu HC, Hsu KH, Yen CJ, Chan SH, Huang W, Su IJ A clustered ground-glass hepatocyte pattern represents a new prognostic marker for the recurrence of hepatocellular carcinoma after surgery. Cancer.

[9] Mathai AM, Alexander J, Kuo FY, Torbenson M, Swanson PE, Yeh MM (2013). Type II ground-glass hepatocytes as a marker of hepatocellular carcinoma in chronic hepatitis B. Hum Pathol.

[10] Wang HC, Wu HC, Chen CF, Fausto N, Lei HY, Su IJ (2003). Different types of ground glass hepatocytes in chronic hepatitis B virus infection contain specific pre-S mutants that may induce endoplasmic reticulum stress. Am J Pathol.

[11] Wang HC, Chang WT, Chang WW, Wu HC, Huang W, Lei HY, Lai MD, Fausto N, Su IJ (2005). Hepatitis B virus pre-S2 mutant upregulates cyclin A expression and induces nodular proliferation of hepatocytes. Hepatology.

[12] Wu HC, Tsai HW, Teng CF, Hsieh WC, Lin YJ, Wang LH, Yuan Q, Su IJ (2014). Ground-glass hepatocytes co-expressing hepatitis B virus X protein and surface antigens exhibit enhanced oncogenic effects and tumorigenesis. Hum Pathol.

[13] Hoshida Y, Toffanin S, Lachenmayer A, Villanueva A, Minguez B, Llovet JM (2010). Molecular classification and novel targets in hepatocellular carcinoma: recent advancements. Semin Liver Dis.

[14] Babinet C, Farza H, Morello D, Hadchouel M, Pourcel C (1985). Specific expression of hepatitis B surface antigen (HBsAg) in transgenic mice. Science.

[15] Wursthorn K, Lutgehetmann M, Dandri M, Volz T, Buggisch P, Zollner B, Longerich T, Schirmacher P, Metzler F, Zankel M, Fischer C, Currie G, Brosgart C, Petersen J (2006). Peginterferon alpha-2b plus adefovir induce strong cccDNA decline and HBsAg reduction in patients with chronic hepatitis B. Hepatology.

[16] Moucari R, Mackiewicz V, Lada O, Ripault MP, Castelnau C, Martinot-Peignoux M, Dauvergne A, Asselah T, Boyer N, Bedossa P, Valla D, Vidaud M, Nicolas-Chanoine MH, Marcellin P (2009). Early serum HBsAg drop: a strong predictor of sustained virological response to pegylated interferon alfa-2a in HBeAg-negative patients. Hepatology.

[17] Lucifora J, Xia Y, Reisinger F, Zhang K, Stadler D, Cheng X, Sprinzl MF, Koppensteiner H, Makowska Z, Volz T, Remouchamps C, Chou WM, Thasler WE, Huser N, Durantel D, Liang TJ (2014). Specific and nonhepatotoxic degradation of nuclear hepatitis B virus cccDNA. Science.

[18] Kremsdorf D, Soussan P, Paterlini-Brechot P, Brechot C (2006). Hepatitis B virus-related hepatocellular carcinoma: paradigms for viral-related human carcinogenesis. Oncogene.

[19] Knodell RG, Ishak KG, Black WC, Chen TS, Craig R, Kaplowitz N, Kiernan TW, Wollman J (1981). Formulation and application of a numerical scoring system for assessing histological activity in asymptomatic chronic active hepatitis. Hepatology.

[20] Brunt EM, Janney CG, Di Bisceglie AM, Neuschwander-Tetri BA, Bacon BR (1999). Nonalcoholic steatohepatitis: a proposal for grading and staging the histological lesions. Am J Gastroenterol.

[21] Chen Y, Sze J, He ML (2004). HBV cccDNA in patients' sera as an indicator for HBV reactivation and an early signal of liver damage. World J Gastroenterol.

[22] Shen FC, Su IJ, Wu HC, Hsieh YH, Yao WJ, Young KC, Chang TC, Hsieh HC, Tsai HN, Huang W (2009). A pre-S gene chip to detect pre-S deletions in hepatitis B virus large surface antigen as a predictive marker for hepatoma risk in chronic hepatitis B virus carriers. J Biomed Sci.

