# Supplementary material for: Resistance of ground glass hepatocytes to oral antivirals in chronic hepatitis B patients and implication for the development of hepatocellular carcinoma
Source: Oncotarget. 2016 Mar 26;7(19):27724–34. doi: 10.18632/oncotarget.8388 (PMC5053683; doi:10.18632/oncotarget.8388)
Supplement: Supplementary file 1 [file oncotarget-07-27724-s001.pdf]

SUPPLEMENTARY FIGURES AND TABLES

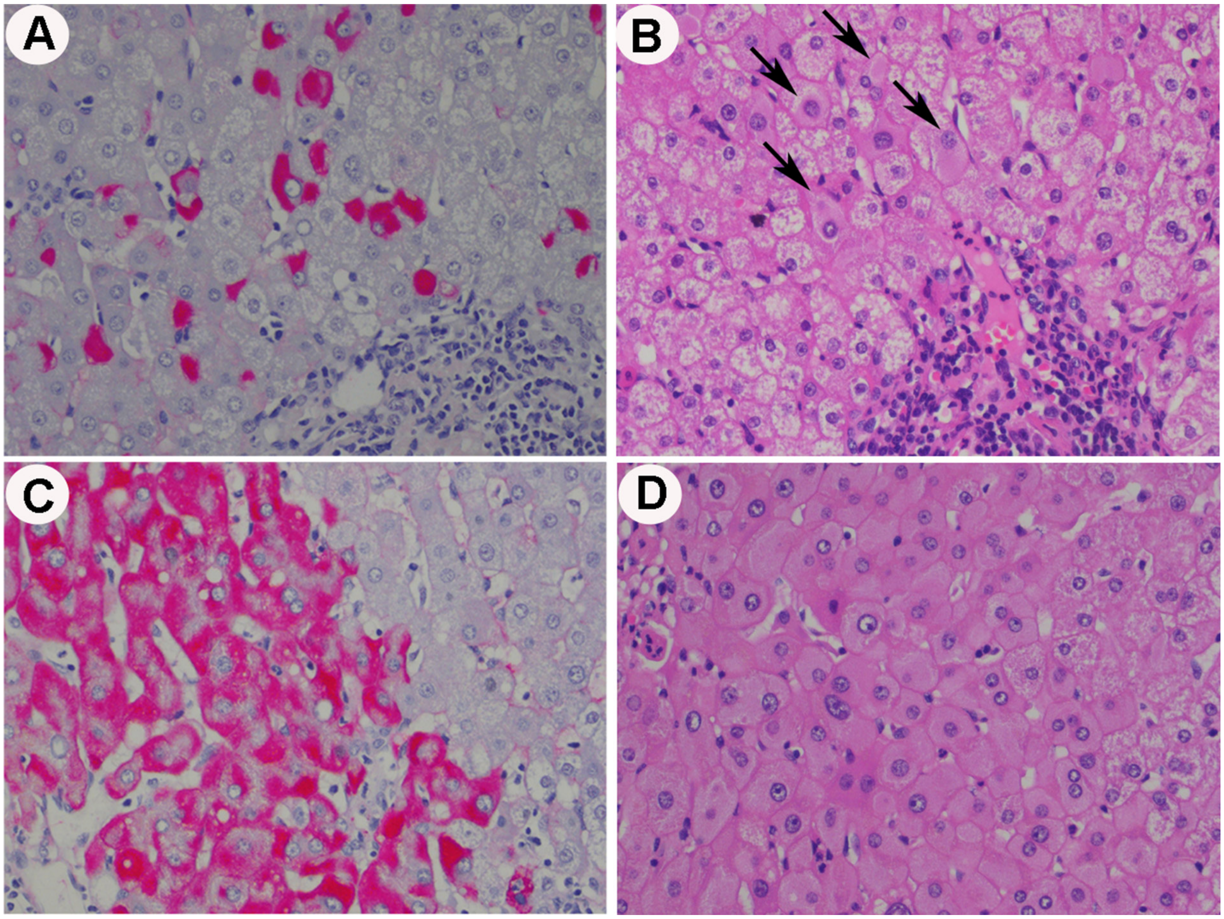

Supplementary Figure S1: Representative histological pictures of non-tumorous liver from a patient without pre-surgical anti-HBV treatment. Type I GGH **A.** type II GGH **C.** in HBsAg stain and corresponding images of the type I GGH (arrows) **B.** and type II GGH **D.** in H&E stain were shown (200X).

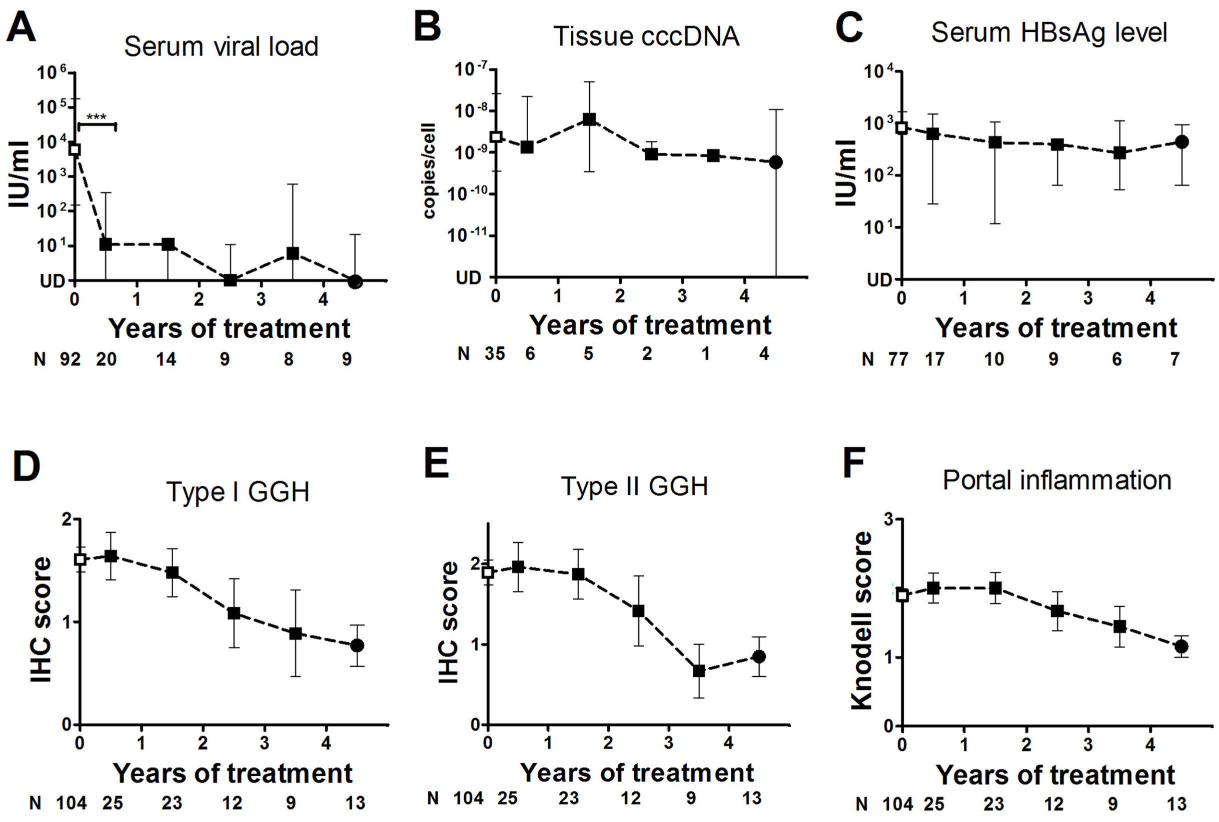

Supplementary Figure S2: The estimated kinetics of serum viral load **A.** tissue cccDNA **B.** serum HBsAg level **C.** type I GGH **D.** and type II GGH **E.** and portal inflammation **F.** in response to the duration of pre-surgical anti-HBV treatment in HCC patients. The medians or means of each marker in patients with the same duration of treatment were plotted in terms of years as closed squares and patients with more than 4 years of treatment were lumped together as the closed circle (open square: non-treated cases for comparison). (Median with interquartile range in A-C and mean with standard error of the mean in D-F; GGH, ground glass hepatocyte; ***, $P<0.001$).

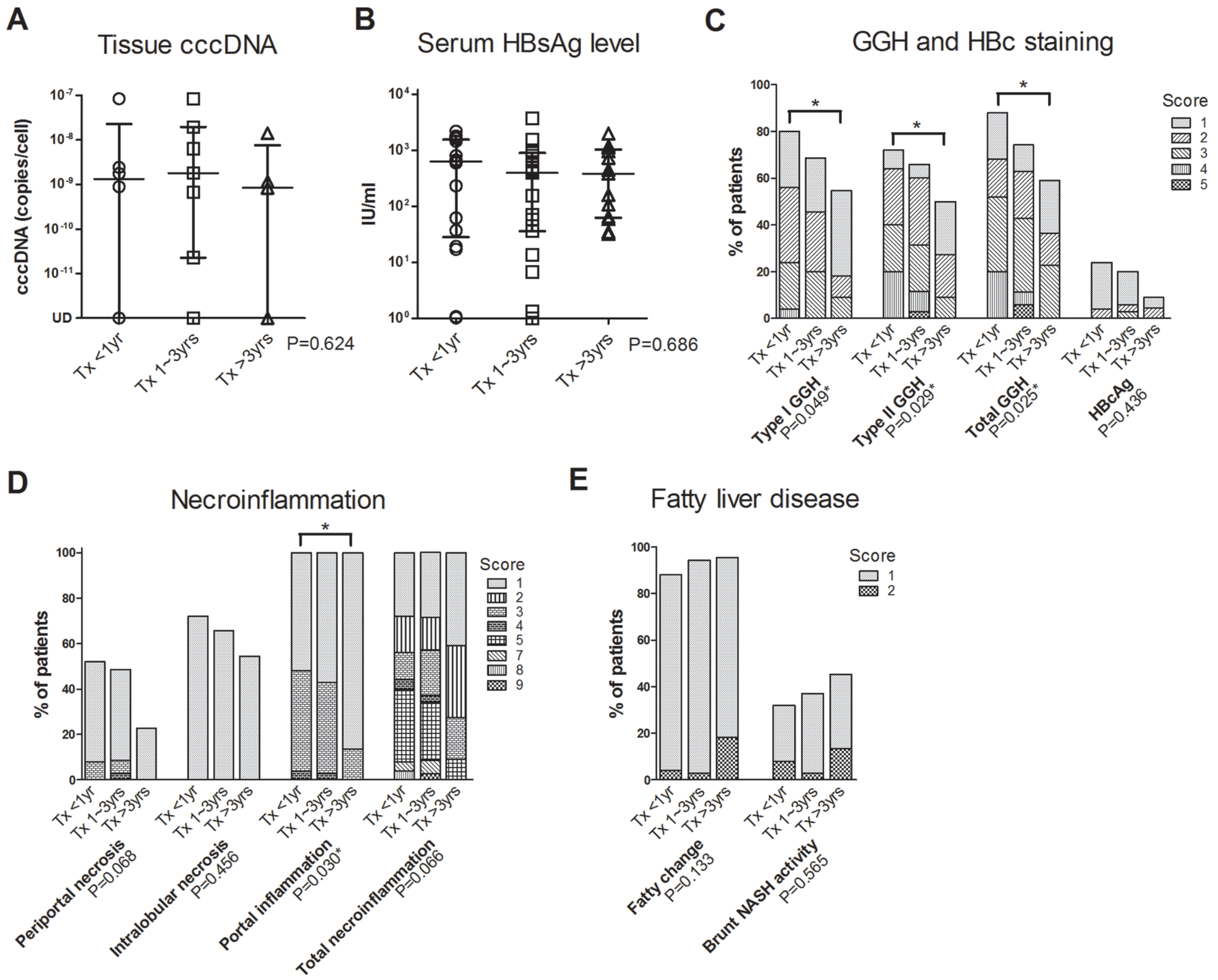

Supplementary Figure S3: The effects of anti-HBV treatment duration on tissue cccDNA **A.** serum HBsAg level **B.** and non-tumorous liver pathologic findings, including viral protein expression **C.** Knodell necroinflammatory score **D.** and fatty liver disease **E**. Patients were separated into 3 groups: short duration for less than one year, medium duration for one to 3 years and long duration for more than 3 years of anti-viral treatment. (Lines in A and B at median with interquartile range. GGH, ground glass hepatocyte; *, $P<0.05$).

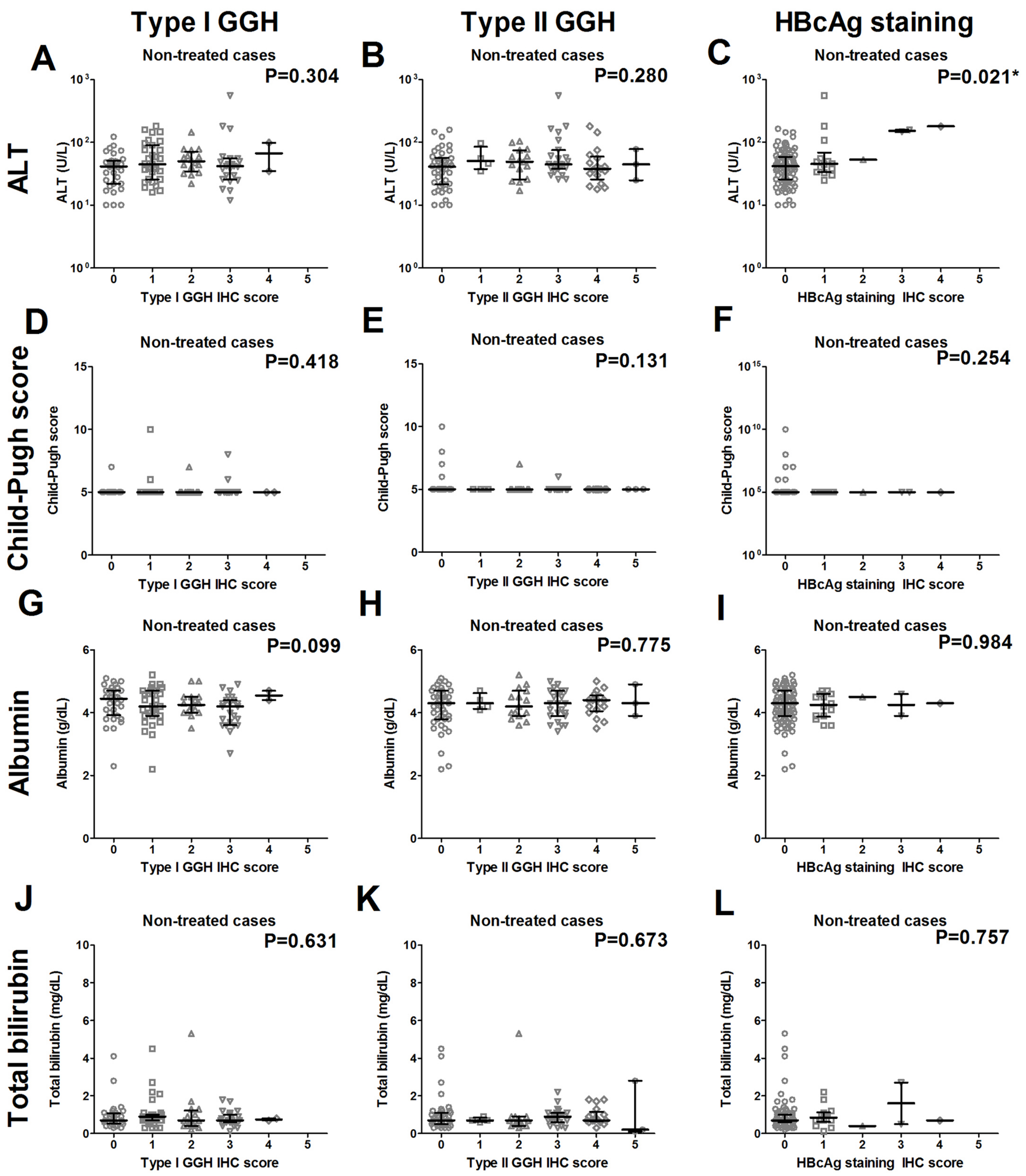

Supplementary Figure S4: The relationships between intrahepatic viral protein expression and alanine aminotransferase (ALT) **A-C.** Child-Pugh score **D-F.** albumin **G-I.** or total bilirubin **J-L.** in non-treated cases. (Lines at median with interquartile range).

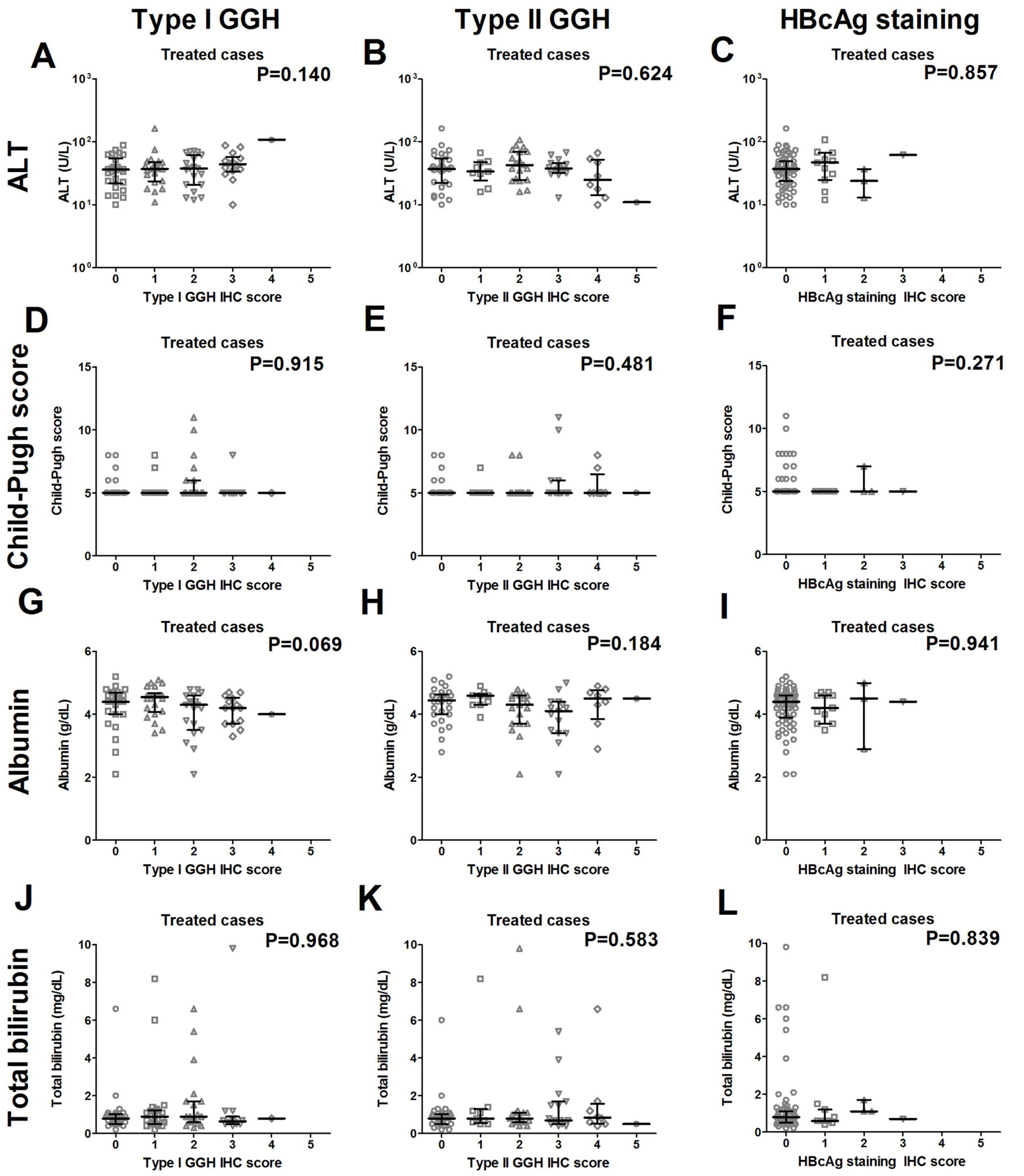

Supplementary Figure S5: The relationships between intrahepatic viral protein expression and alanine aminotransferase (ALT) **A-C.** Child-Pugh score **D-F.** albumin **G-I.** or total bilirubin **J-L.** in treated cases. (Lines at median with interquartile range).

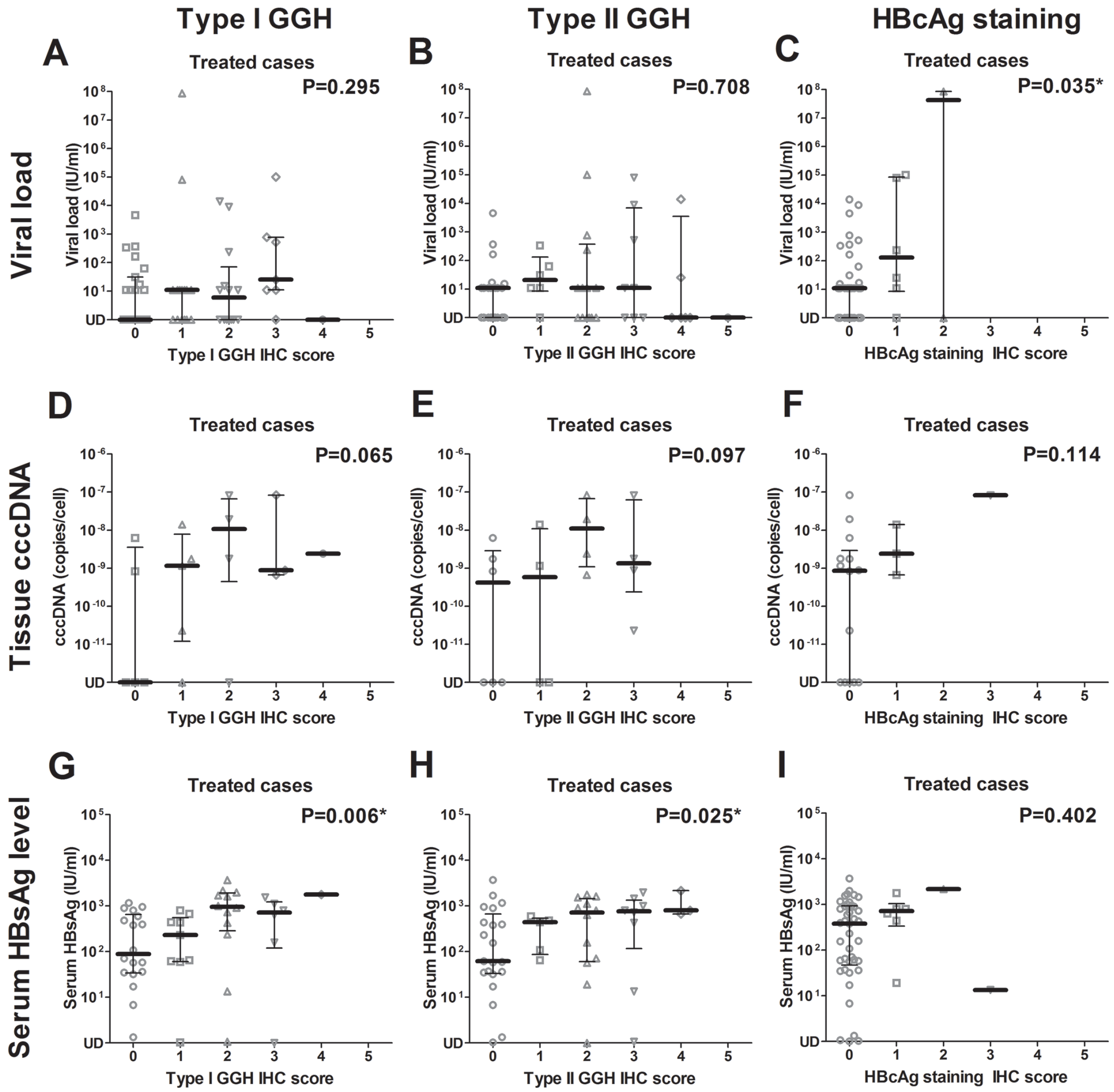

Supplementary Figure S6: The relationships between intrahepatic viral protein expression and serum viral load **A-C.** intrahepatic cccDNA **D-F.** or serum HBsAg level **G-I.** in treated cases. (Lines at median with interquartile range).

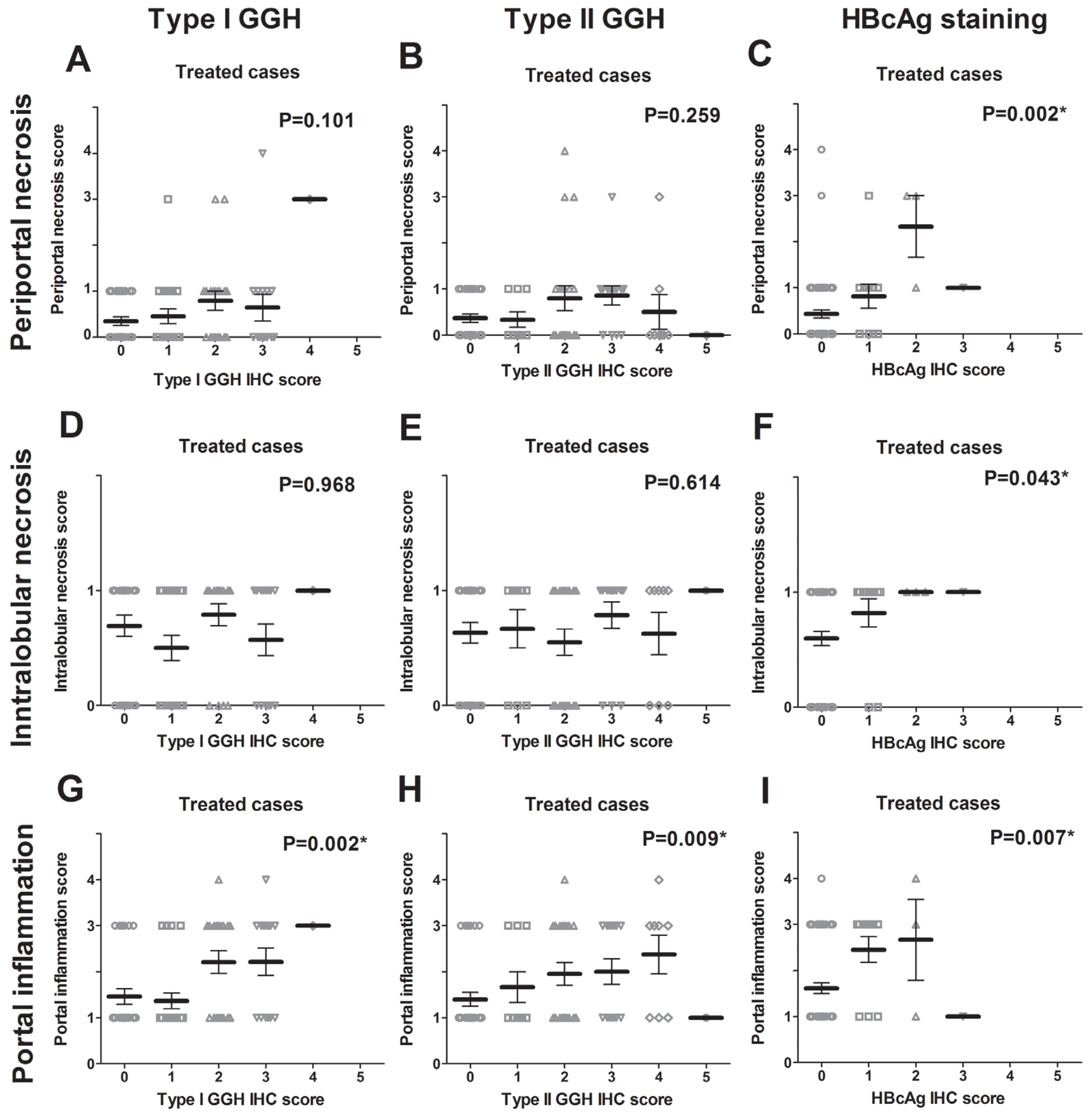

Supplementary Figure S7: The relationships between intrahepatic viral protein expression and periportal necrosis **A-C.** intralobular necrosis **D-F.** or portal inflammation **G-I.** in treated cases. (Lines at mean with standard error of the mean).

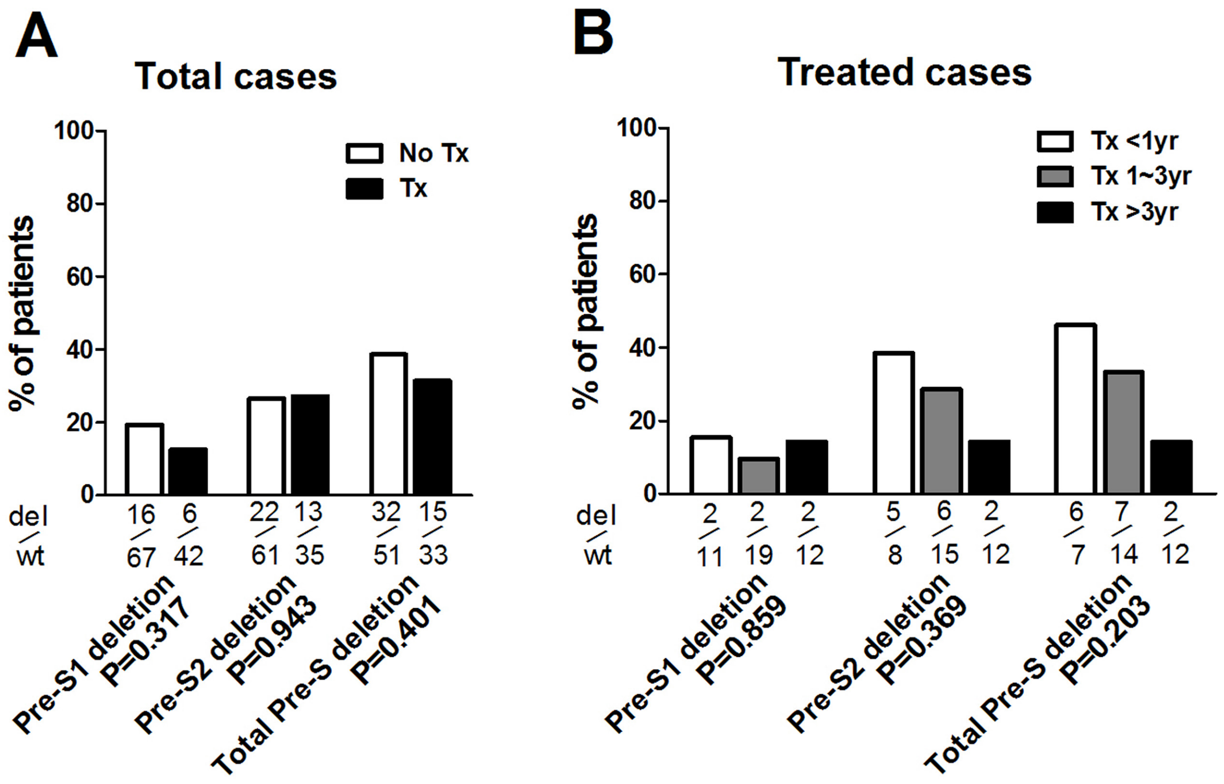

Supplementary Figure S8: Comparisons of serum pre-S mutation status between non-treatment group (no Tx) and treatment group (Tx) **A.** and among patients with various durations of treatment **B**.

Supplementary Table S1: Clinicopathological data of 31 patients with liver biopsy specimens taken week 0 and week week 48 of oral nucleos(t)ide analogue treatment

Variables	Week 0	Week 48	P
Clinical and laboratory data			
ALT (U/L)#	165 (100~267)	33 (27~47)	<0.001*
Albumin (g/dL)#	4.4 (4.2~4.7)	4.4 (4.2~4.7)	0.243
Total bilirubin (mg/dL)#	0.9 (0.6~1.1)	0.8 (0.5~1.1)	0.124
Serum HBeAg (-/total)	11/31 (35.5%)	15/31 (48.4%)	0.046*
Viral load (IU/ml)#	$7.63x10^7$ ($1.27x10^7$~$2.2x10^7$)	51.4 (<20~$1.54x10^3$)	<0.001*
HBsAg level (IU/ml)#	$1.19x10^4$ ($5.95x10^3$~$6.09x10^4$)	$1.56x10^3$ ($6.62x10^2$~$5.07x10^3$)	<0.001*
Pathologic parameters			
Type I GGH (score)$	0.87 +/- 1.09	0.97 +/- 1.02	0.527
Type II GGH (score)$	0.29 +/- 0.86	0.58 +/- 1.26	0.077
Total GGH (score)$	0.97 +/- 1.22	1.29 +/- 1.44	0.101
HBcAg (score)$	2.84 +/- 1.68	1.03 +/- 1.60	<0.001*
Periportal necrosis (score)$	1.13 +/-1.61	0.45 +/- 0.81	0.003*
Intralobular necrosis (score)$	1.19+/-0.75	0.87 +/- 0.56	0.085
Portal inflammation (score)$	1.97 +/- 1.11	1.45 +/- 1.06	0.028*
Knodell necroinflammation (score)$	4.29 +/- 2.75	2.77 +/- 1.86	0.001*
Knodell fibrosis (score)$	0.87 +/- 0.96	0.90 +/- 1.14	0.815

$, Mean +/- SD; #, Medium (interquartile range). ALT, alanine transaminase. AFP, alpha-fetoprotein.

Supplementary Table S2: Comparison of clinicopathological data between HCC patients with and without pre-surgical anti-HBV therapy at the time point of surgery

Variables	Non-treatment(N=104)	Treatment(N=82)	P
Clinical and laboratory data			
Age (years)$^{\$}$	55.68 +/- 12.17	57.90 +/- 9.20	0.197
Gender: male / female	76/28	62/20	0.695
ALT (U/L)$^{\#}$	43.5 (28.5~60.8)	37.5 (24~52.3)	0.047*
Albumin (g/dL)$^{\#}$	4.3 (3.9~4.7)	4.4 (3.9~4.6)	0.745
Total bilirubin (mg/dL)$^{\#}$	0.7 (0.6~1.0)	0.8 (0.5~1.1)	0.325
Serum HBeAg (-/total)	60/65 (92.3%)	64/76 (84.2%)	0.141
Viral load (IU/ml)$^{\#}$	$5.87x10^3$ (145.5~$1.7x10^5$)	<20 (undetectable~22.3)	<0.001*
HBsAg level (IU/ml)$^{\#}$	825 (66.19~1606.12)	628.44 (46.15~932.06)	0.050
AFP (ng/ml)$^{\#}$	13.32 (5.20~208.63)	9.33 (4.98~36)	0.074
Child-Pugh class: A/B/C	100/3/1	72/8/2	0.099
Non-tumor part pathology			
Type I GGH (score)$^{\$}$	1.35 +/- 1.19	1.29 +/- 1.13	0.811
Type II GGH (score)$^{\$}$	1.79 +/- 1.66	1.56 +/- 1.44	0.376
Total GGH (score)$^{\$}$	1.96 +/- 1.62	1.85 +/- 1.43	0.594
HBcAg (score)$^{\$}$	0.25 +/- 0.66	0.24 +/- 0.58	0.857
Periportal necrosis (score)$^{\$}$	0.81 +/-0.83	0.56 +/- 0.83	0.006*
Intralobular necrosis (score)$^{\$}$	0.75+/-0.44	0.65 +/- 0.48	0.125
Portal inflammation (score)$^{\$}$	1.89 +/- 1.01	1.76 +/- 1.01	0.344
Knodell necroinflammation (score)$^{\$}$	3.45 +/- 1.84	2.96 +/- 1.93	0.047*
Fatty change (score)$^{\$}$	0.95 +/- 0.45	1 +/- 0.38	0.433
Brunt NASH activity (score)$^{\$}$	0.38 +/- 0.55	0.45 +/- 0.63	0.604
Knodell fibrosis (score)$^{\$}$	3.07 +/- 0.99	3.51 +/- 0.92	<0.001*
Tumor part pathology			
Differentiation (W/M/P)	14/68/22	14/57/11	0.170
Multifocal tumor (+/total)	20/104 (19.2%)	16/82 (19.5%)	0.962
Satellite nodule (+/total)	32/104 (30.8%)	14/82 (17.1%)	0.032*
Tumor size (cm)$^{\$}$	5.15 +/- 3.53	3.47 +/- 2.92	<0.001*
Tumor encapsulation (+/total)	10/104 (9.6%)	8/82 (9.8%)	0.974
Vascular invasion (+/total)	54/104 (51.9%)	38/82 (46.3%)	0.450
AJCC stage (I/II/IIIA/IIIB/IIIC/IVA/IVB)	31/49/9/4/9/1/1	31/39/6/2/4/0/0	0.148

$^{\$}$, Mean +/- SD; $^{\#}$, Medium (interquartile range); W/M/P, well/moderately/poorly on the basis of the World Health Organization classification. The satellite nodule was defined according to Rosai and Ackerman's Surgical pathology, 9th edition (page 2844). The presence of multifocal tumors was defined by the presence of another nodule beyond the criteria of a satellite nodule. Encapsulation was defined as a tumor totally confined by a fibrous pseudocapsule. ALT, alanine transaminase. AFP, alpha-fetoprotein.

Supplementary Table S3: Pre-surgical anti-HBV treatment profiles in 82 HCC patients of the treatment group

Oral nucleoside analogue	n	Duration (months)
Entecavir	58	Median: 17.7, range: 0.6-132.0
Lamivudine→Entecavir	8	Median: 40.9, range: 12.6-100.0
Adefovir→Entecavir	4	Median: 37.8, range: 15.3-105.4
Lamivudine→Adefovir→Entecavir	1	61.8
Lamivudine→Adefovir→Peginterferon alfa-2a →Entecavir	1	70.9
Lamivudine→Adefovir	1	51.9
Lamivudine→Adefovir+Lamivudine→Tenofovir+Lamivudine	1	70.5
Adefovir	2	12.0, 20.0
Adefovir+Lamivudine	1	7.0
Lamivudine	5	Median: 12.7, range: 1.0-127.5

Supplementary Table S4: Prognostic significance of clinicopathological indicators, HBsAg expression patterns and HBV serum profiles for the total HCC patients

Factor	LRFS			OS		
	Group	Univariate	Multivariate	Group	Univariate	Multivariate
Serum profiles						
Viral load (IU/ml)	(<10^4, ≥10^4)	0.807		(<10^4, ≥10^4)	0.572	
HBsAg level	(<800, ≥800)	0.142		(<800, ≥800)	0.052	
Pre-S1 mutation	(-,+)	0.041*	NS	(-,+)	0.613	
Pre- S2 mutation	(-,+)	0.381		(-,+)	0.943	
Pre-S1 and S2 mutation	(-,+)	0.235		(-,+)	0.225	
Tumor factor						
Differentiation	(W-M, P)	0.009*	NS	(W-M, P)	0.093	
Multifocal tumors	(-,+)	0.053		(-,+)	0.397	
Satellite nodule	(-,+)	0.001*	NS	(-,+)	0.037*	NS
Size (cm)	(<5, ≥5)	<0.001*	<0.001*	(<5, ≥5)	<0.001*	NS
Encapsulation	(-,+)	0.445		(-,+)	0.140	
Vascular invasion	(-,+)	0.010*	NS	(-,+)	0.002*	NS
AJCC stage	(I, ≥II)	<0.001*	<0.001*	(II, ≥III)	<0.001*	<0.001*
Non-tumor liver pathology						
Knodell necroinflammatory score	(≤5,≥6)	0.028*	NS	(≤5,≥6)	0.014*	<0.001*
Knodell fibrosis score	(≤2,≥3)	0.164		(≤2,≥3)	0.231	
Fatty change	(<5%,≥5%)	0.647		(<5%,≥5%)	0.371	
Type I GGH score	(0-2, 3-4)	0.100		(0-2, 3-4)	0.197	
Type II GGH score	(0-2, 3-4)	0.003*	<0.001*	(0-2, 3-4)	0.033*	0.022*
HBcAg score	(0-2, 3-4)	0.002*	NS	(0-1, 2-4)	0.022*	NS

NS, not significant; LRFS, local recurrence-free survival; OS, overall survival; (W, M, P), (well, moderately, Poorly-differentiated on the basis of the World Health Organization classification). The satellite nodule was defined according to Rosai and Ackerman's Surgical pathology, 9th edition (page 2844). The presence of multifocal tumors was defined by the presence of another nodule beyond the criteria of a satellite nodule. Encapsulation was defined as a tumor totally confined by a fibrous pseudocapsule. * P<0.05.
